# Biological Evaluation and In Vitro Characterization of ADME Profile of In-House Pyrazolo[3,4-*d*]pyrimidines as Dual Tyrosine Kinase Inhibitors Active against Glioblastoma Multiforme

**DOI:** 10.3390/pharmaceutics15020453

**Published:** 2023-01-30

**Authors:** Federica Poggialini, Chiara Vagaggini, Annalaura Brai, Claudia Pasqualini, Emmanuele Crespan, Giovanni Maga, Cecilia Perini, Noemi Cabella, Lorenzo Botta, Francesca Musumeci, Maria Frosini, Silvia Schenone, Elena Dreassi

**Affiliations:** 1 Department of Biotechnology, Chemistry and Pharmacy (DBCF), University of Siena, 53100 Siena, Italy; 2 Institute of Molecular Genetics (IGM), CNR “Luigi Luca Cavalli-Sforza”, 27100 Pavia, Italy; 3 Department of Ecological and Biological Sciences, University of Tuscia, Via S.C. De Lellis s.n.c., 01100 Viterbo, Italy; 4 Department of Pharmacy, University of Genoa, 16132 Genoa, Italy; 5 Department of Life Sciences, University of Siena, 53100 Siena, Italy

**Keywords:** glioblastoma, tyrosine kinase, c-Src/Abl dual inhibitors, pyrazolo[3,4-*d*]pyrimidines, ADME, antiproliferative activity

## Abstract

The therapeutic use of tyrosine kinase inhibitors (TKIs) represents one of the successful strategies for the treatment of glioblastoma (GBM). Pyrazolo[3,4-*d*]pyrimidines have already been reported as promising small molecules active as c-Src/Abl dual inhibitors. Herein, we present a series of pyrazolo[3,4-*d*]pyrimidine derivatives, selected from our in-house library, to identify a promising candidate active against GBM. The inhibitory activity against c-Src and Abl was investigated, and the antiproliferative profile against four GBM cell lines was studied. For the most active compounds endowed with antiproliferative efficacy in the low-micromolar range, the effects toward nontumoral, healthy cell lines (fibroblasts FIBRO 2-93 and keratinocytes HaCaT) was investigated. Lastly, the in silico and in vitro ADME properties of all compounds were also assessed. Among the tested compounds, the promising inhibitory activity against c-Src and Abl (*K*_i_ 3.14 µM and 0.44 µM, respectively), the irreversible, apoptotic-mediated death toward U-87, LN18, LN229, and DBTRG GBM cell lines (IC_50_ 6.8 µM, 10.8 µM, 6.9 µM, and 8.5 µM, respectively), the significant reduction in GBM cell migration, the safe profile toward FIBRO 2-93 and HaCaT healthy cell lines (CC_50_ 91.7 µM and 126.5 µM, respectively), the high metabolic stability, and the excellent passive permeability across gastrointestinal and blood–brain barriers led us to select compound **5** for further in vivo assays.

## 1. Introduction

According to the World Health Organization (WHO), glioblastoma multiforme (GBM) is classified as the most aggressive form of primary brain cancer (grade IV), associated with poor prognosis, high morbidity, and mortality [1]. Among GBM patients, the median survival time is typically no longer than 1 year from the diagnosis, and <3% of cases have a 5-year survival rate. Responsible for 18% of brain tumors, GBM is one of the most common primary tumors of the central nervous system in adults [2]. Histologically, GBM is typed by high microvascularity and necrotic areas, typical signs of the high proliferation, invasiveness, and angiogenesis of the tumor, demanding nutrients and oxygen for its development. The current standard treatments of GBM are represented by surgical resection, followed by a combination of radiotherapy and chemotherapy; despite these massive and intense treatments, the prognosis remains inauspicious [3]. Failure in GBM therapy is mostly represented by the lack of drugs able to hit effective molecular targets, overcome the blood–brain barrier, and escape from multidrug resistance mechanisms occurring during the therapy. Therefore, novel and more effective pharmacological approaches are urgently needed to improve the prognosis of GBM patients [4]. Genomic studies highlighted the aberrant activation of receptor tyrosine kinases (RTKs) over different types of cancers, including GBM. A common feature of GBM has been identified by transcriptional analyses in the downregulation of TP53, RB1, and RTKs/Ras/PI3K pathways, recognizing these as fundamental requirements for the pathogenesis of GBM. Moreover, the massive activation of RTKs leads to the downregulation of the downstream signaling cascade resulting in the activation of both ERK and MAPK pathways and non-receptor tyrosine kinases (TKs), such as Src and Abl. Src family kinases regulate cell proliferation, survival, and angiogenesis and play an essential role in cancer progression and diffusion [5]. Additionally, the upregulation of the Abelson (Abl) family members is a trademark of GBM, involved in tumorigenesis, proliferation, and migration [6,7]. Thus, targeted therapy toward activated Src and Abl might represent a favorable strategy to counteract the high proliferation rate and invasion of GBM. In the last few decades, great attention has been paid to the implication of TKs in glioma biology and development of novel small-molecule compounds able to target and inhibit these molecular targets. Although monotherapy with Src inhibitors, such as dasatinib and bosutinib, resulted unsatisfactory for recurrent GBM, TKs remain one of the most promising targets to arrest cancer invasion [4]. In this context, several pyrazolo[3,4-*d*]pyrimidines have been studied and developed by our research group as ATP-competitive TKIs. Several compounds showed potent antitumor activities, inducing apoptosis and downregulating cell proliferation, in several cancer cell lines, including solid tumors such as osteosarcoma [8], neuroblastoma [9], GBM [1,5,10], and mesothelioma [11], as well as hematological tumors such as leukemia and Burkitt lymphoma [12,13]. In vivo data, obtained from xenograft mouse models, supported the antitumor activity of these small molecules against neuroblastoma, leukemia, and GBM [14]. Compound **1** was previously reported by us as a potent c-Src/Abl dual inhibitor with favorable *K*_i_ values and micromolar efficacy against the U-87 GBM cell line [10]. The ADME profile was characterized by suboptimal aqueous solubility and good passive permeability across the gastrointestinal (GI) and blood–brain barrier (BBB) membranes, but metabolic stability was lower than 80%. With the purpose of identifying molecules with the right balance between efficacy against GBM and suitable pharmacokinetic properties, we selected a series of pyrazolo[3,4-*d*]pyrimidines from our internal library. We performed structure–activity relationship (SAR) studies along with biological and ADME evaluations to select a promising molecule active as a TKI against GBM. The compounds were characterized by heterogeneous chemical features at the N1, C4, and C6 positions. First, a cell-free assay was set up to determine the *K*_i_ values against c-Src and Abl, followed by the assessment of the antiproliferative activity against four GBM cell lines (U-87, LN18, LN229, and DBTRG). Furthermore, the cytotoxic profile was established on two nontumoral, healthy cell lines (fibroblasts FIBRO 2-93 and keratinocytes HaCaT) for the most active compounds of the series. Lastly, the in silico and in vitro ADME profile was investigated for all derivatives. On the basis of the biological and ADME studies, compound **5** resulted as the most promising pyrazolo[3,4-*d*]pyrimidine derivative of the panel. Its favorable inhibitory activity against c-Src/Abl, its promising antitumor efficacy against all GBM cell lines tested, and its safe cytotoxic profile led us to select compound **5** for further in vitro assays. Its ability to elicit an irreversible cytotoxic effect, arrest cellular migration, and induce apoptotic-mediated cell death was assessed. Moreover, the high metabolic stability and satisfactory passive permeability across the GI and BBB membranes suggest that compound **5**, with appropriate formulation studies to overcome solubility issues, could represent a strong candidate for future in vivo PK studies.

## 2. Materials and Methods

### 2.1. Drugs and Materials

Compounds herein reported were previously synthesized and reported by us [12,15,16,17,18,19]. The NMR characterizations, chemical names, and structures are presented in the Supplementary Materials (Chemistry chapter and Scheme S1). All reagents, solvents, and materials were purchased from Sigma Aldrich S.r.l. (Milan, Italy) and Carlo Erba Reagents S.r.l (Milan, Italy). Cell culture mediums, fetal bovine serum (FBS), L-glutamine, and penicillin–streptomycin were purchased from Euroclone S.p.A. (Milan, Italy). Human glioblastoma multiforme cell lines (U-87, LN18, LN229, and DBTRG) and the normal human dermal fibroblast (FIBRO 2-93) cell line were purchased from American Type Culture Collection (ATCC) (Manassas, VA, USA). Human keratinocytes (HaCaT) were kindly donated by Professor Federica Pessina from the Department of Molecular and Developmental Medicine (University of Siena, Italy). All the compounds were dissolved in dimethyl sulfoxide (DMSO) as a 20 mM stock solution immediately before use and diluted to final desired concentration with appropriate cell culture medium (see below).

### 2.2. Enzymatic Assay

Active recombinant kinases and specific peptide substrates were purchased from Promega (Madison, WI, USA). The reactions were carried out in accordance with the manufacturer’s instructions, with minor modifications. The experiments were run using all substrates at least twice the concentration of apparent *K*_m_. Src reactions were carried out with 500 µM Src-peptide (KVEKIGEGTYGVVYK), 100 µM ATP, and 0.00087% NP-40; Abl reactions were performed with 50 µM Abltide (EAI-YAAPFAKKK), 30 µM ATP, and 0.00087% NP-40, and T315I reactions were carried out with 100 µM Abltide, 60 µM ATP, and 0.0013% NP-40. All reactions were run out at 30 °C for 10 min with 10–50 ng of enzyme and 10 µL of 10% DMSO. Protein low-binding tubes were used to prevent peptide adsorption onto the plastic surface. To detect kinase activity, the ADP-Glo Kinase Assay (Promega, Madison, WI, USA) was used according to the manufacturer’s protocol. Into white 384-well plates, reactions were stopped for 50 min at RT by adding 10 µL of ADP-Glo Reagent (Promega, Madison, WI, USA). After 30 min, 20 µL of Detection Reagent (Promega, Madison, WI, USA) was added, and the reaction was read using a GloMax Discover microplate reader (Promega, Madison, WI, USA). Data were plotted using GraphPad Prism 5.04 (GraphPad Software Inc., San Diego, CA, USA), and ID_50_ values were calculated according to Equation (1).

ν = V/(1 + (I/ID_50_)), (1)

where ν represents the measured reaction velocity, V is the apparent maximal velocity without the inhibitor, I is the inhibitor concentration, and ID_50_ is the 50% inhibitory dose. Compounds analyzed were considered to act as ATP-competitive inhibitors. Thus, *K*_i_ values were calculated using Equation (2).

*K*_i_ = (ID_50_/(1 + KmATP/[SATP]))/(1 + Kmpep/[Spep]), (2)

where *K*_i_ represents the affinity of the inhibitor for the enzyme, S is the ATP concentration, and Km is the affinity of ATP calculated according to Michaelis–Menten equation.

### 2.3. Cell Culture, Antiproliferative Effects, and Cytotoxicity on Cell-Based Assays

Cell-based assays were performed using four GBM cell lines, i.e., U-87, LN18, LN229, and DBTRG, cultured in Dulbecco’s modified Eagle medium (DMEM) (U-87, LN18, and LN229) or Roswell Park Memorial Institute medium (RPMI 1640) (DBTRG). Both media were implemented with 10% fetal bovine serum (FBS), 2 mM L-glutamine, and 10,000 units/mL penicillin/streptomycin at 37 °C in a 5% CO_2_ atmosphere. To evaluate the antiproliferative activity of the compounds reported as IC_50_ (i.e., drug concentration that caused 50% of cell growth inhibition), cells were seeded at a density of 1 × 10^4^ in 96-well plates and, after 24 h of incubation, treated with increasing concentrations of compounds for an additional 72 h. The final concentration of DMSO used never exceeded 0.5% *v*/*v*, and appropriate controls were always performed in each assay. To assess the potential cytotoxic profile of the three most promising and active compounds toward nontumoral cells, reported as CC_50_ (i.e., concentration that reduced the proliferation of cells by 50%), further investigations were conducted using healthy fibroblasts FIBRO 2-93 and keratinocytes HaCaT. These were cultured in DMEM supplemented with 10% FBS, 2 mM L-glutamine, and 10,000 units/mL penicillin/streptomycin at 37 °C in a 5% CO_2_ atmosphere. The protocol used to assess the cytotoxic effect on both healthy cell lines was the same described above for the antiproliferative assay. For both the antiproliferative and the cytotoxic assays, 3-(4,5-dimethylthiazol-2-yl)-2,5-diphenyltetrazolium bromide (MTT) was used to assess cell viability as previously described [20]. Briefly, 150 μL of MTT (5 mg/mL in FBS-free culture medium) was added to each well, and the plates incubated for 4 h in a 5% CO_2_ atmosphere at 37 °C. Afterward, formazan violet crystals were solubilized in 150 μL of isopropanol, and the absorbance was quantified at 570 nm using a Multiskan SkyHigh Microplate Spectrophotometer (ThermoFisher, Waltham, MA, USA). Cell viability was expressed as a percentage of DMSO-treated cells (controls), taken as 100%. IC_50_ and CC_50_ values were calculated by fitting data to a nonlinear regression analysis (sigmoidal log concentration vs. normalized response curve; GraphPad Prism 5.04 software, GraphPad Software Inc., San Diego, CA, USA) using at least six points in a concentration range from 0% to 100% of the studied effects (total widest range 0.01–1000 µM). To support MTT data, cytotoxicity assays was always paralleled by a careful observation of cells under a phase-contrast light microscope, as described in USP 28 (United States Pharmacopeia edition 2005) as an alternative method [21,22,23]. This analysis was performed by an expert operator blind to the treatment, and exemplificative photos representing the effects of compound **5** on LN229 and DBTRG cells are reported in Figures S2 and S3 (Supplementary Materials).

### 2.4. In Vitro ADME Assays

#### 2.4.1. HPLC/UV–MS Method

UV/LC–MS chromatographic analyses were carried out using an Agilent 1100 LC/MSD VL system (G1946C) purchased from Agilent Technologies (Palo Alto, CA, USA). Chromatographic separations were achieved at room temperature (RT) using a Phenomenex Kinetex C18-100 column (150 mm × 4.6 mm, 5 µm) and gradient elution consisting of a solution of solvent A (H_2_O) and solvent B (ACN), both acidified with 0.1% *v*/*v* formic acid (FA). The analysis began with 5% B (t = 0–1 min), increased to 95% (t = 1–10 min), maintained at 95% (t = 10–19 min), and finally returned to 5% solvent A. The analyses were performed at a flow rate of 0.6 mL/min, UV detection was monitored at 254 nm, and spectra were acquired over the scan range of *m*/*z* 100–1000 in both positive and negative modes.

#### 2.4.2. Parallel Artificial Membrane Permeability Assay (PAMPA)

The DMSO stock of each compound was diluted with phosphate buffer (PBS 25 mM, pH 7.4) to a final concentration of 500 µM in order to obtain the “donor”. For GI and BBB permeability, filters were coated with 10 µL of 1% *w*/*v* dodecane phosphatidylcholine solution or 5 µL of 10% *w*/*v* CHCl_3_/dodecane brain polar lipid solution. The donor (150 µL) was poured over the artificial membranes on the filter plate. DMSO/PBS (300 µL, 1:1 *v*/*v*) was then added to the acceptor wells. After assembling the sandwich plates, the experiments were run for 5 h at room temperature (RT). Lastly, the amount of compound passed though the artificial membranes was determined using the UV/LC–MS method described above. Apparent permeability (Papp) and membrane retention (MR%) were determined as previously described [24,25].

#### 2.4.3. Metabolic Stability Assay

Each DMSO compound solution was incubated for 1 h at 37 °C in the presence of 500 µL of PBS (25 mM, pH 7.4), human liver microsomal (HLM) proteins (0.2 mg/mL), and 10 mM NADPH solution in MgCl_2_ (48 mM). The reactions were stopped with 1.0 mL of cold ACN. The samples were centrifuged (4000 rpm for 10 min), and the supernatants were collected, dried under N_2_ flow, and suspended in 100 µL of methanol (MeOH). Parent drugs and metabolites were determined using the previously described HPLC/UV–MS method. The percentage metabolic stability was calculated by comparing the metabolized and unmetabolized compounds.

### 2.5. Reversible/Irreversible Effect on LN229 Cell Death

To check if cytotoxic effects of compound **5** were reversible or irreversible, LN229 cells were seeded and treated as previously described. After 24 h of incubation with increasing concentration of compound **5** (0.1–1–10–100 µM), plates were washed with PBS and cultured in drug-free DMEM with 10% FBS for a further period of 48 h [20]. Afterward, the MTT assay was performed to assess cell viability as previously described.

### 2.6. Cell-Cycle Analysis

Flow cytometry was used to investigate the apoptotic effect of compound **5** on LN229 and DBTRG cells. These were seeded in a six-well plate (5 × 10^5^ cells/well) and treated with compound **5** at the respective IC_50_ values concentrations for 72 h. Afterward, the cells were treated as previously reported [26]. Red fluorescence (DNA) was detected through a 563–607 nm bandpass filter (FL2 channel, 10^4^ cells/sample) using an FACScan flow cytometer coupled with Cell Quest software v.3.0, (both purchase from BD Biosciences, San Jose, CA, USA), and the latter was used to calculate the percentage of cells in the different phases of the cell cycle.

### 2.7. T-Scratch Assay

To investigate the inhibitory effect of compound **5** on cell migration, LN229 cells (8 × 10^4^ cells/well) were seeded in 24 well-plate such that they were at 90% confluence the following day, at which point a scratch was made in the cell layer using a sterile p200 pipet tip. After washing the cells with warm PBS to remove the detached cells, 500 µL of fresh DMEM containing 1% FBS and increasing concentrations (0.25–0.5–2.5–5 µM) of compound **5** were added to each well. The scratched area was observed and photographed at 10× magnification after 0–24–48–72 h (see Figure S5, Supplementary Materials). Finally, the size of the area covered by cells at selected timepoints was obtained using ImageJ software (National Institute of Health, Bethesda, MD, USA, 1.37v), and the percentage cellular migration was plotted as percentage wound closure using GraphPad Prism version 5.04 (GraphPad Software Inc., San Diego, CA, USA).

### 2.8. Data Analysis

Data are reported as the mean ± standard deviation (SD) of at least three independent experiments. Statistical analysis was performed using two-way ANOVA followed by Tukey’s multiple comparison test (reversible/irreversible effect on LN229 cell death, T-scratch assay) and Student’s *t*-test for unpaired samples (cell-cycle analysis) as appropriate.

## 3. Results and Discussion

### 3.1. In Vitro Biological Evaluation

#### 3.1.1. Enzymatic Assay

GBM is a highly aggressive tumor with low expectations of recovery. Today, the standard treatment for GBM is surgical ablation, combined with and/or followed by radiotherapy and chemotherapy. The Src and Abl families play a crucial role in carcinogenesis, angiogenesis, and metastasis, and contribute to the development of the mechanisms of resistance, particularly in GBM, in which they are highly upregulated [5,6,7]. On the basis of these factors, the development of small molecules that act as dual Src/Abl inhibitors with improved pharmacokinetic properties, especially the ability to reach the target beyond the BBB, represents one successful strategy to attack GBM on several fronts while targeting the molecular mechanisms implicated in resistance. Thus, to investigate the structure–activity relationship (SAR) of the selected compounds, a cell-free enzymatic assay was used to assess the affinity toward c-Src and Abl, as shown in Table 1. As already reported [10], compound **1** inhibited both enzymes with *K*_i_ values in the micromolar and low-micromolar range (3 µM and 0.37 µM against c-Src and Abl, respectively). A wide-ranging SAR study on this class of derivatives suggested that the presence of a benzylamine moiety (**3**, **4**, **5**, **6**, **7**, **9**, and **11**) in the R_2_ position resulted in more active derivatives than cyclopropylamine (**2**), aniline (**12**), or phenethylamine ones (**8** and **10**). Among the first group of compounds, with respect to parent compound **1**, an improvement in the inhibitory efficacy against c-Src was achieved thanks to the introduction of a fluorine atom in the ortho (**3**), para (**4**) [16], or meta (**11**) [19] position of the benzylamine group. These modifications led to a significant reduction in the inhibitory efficacy against Abl when compared to that of reference compound **1**. The introduction of a fluorine (**6**) [16] or a chlorine atom (**7**) [17] in the R position resulted in promising derivatives with *K*_i_ values in the low-micromolar range. Compound **5** did not show substantial changes in c-Src/Abl inhibition, despite having one more fluorine atom than **1**. Lastly, the introduction of a morpholine ethanethiol moiety (**9**) in R_3_ provoked a favorable improvement in the inhibition of Abl, but no significant differences against c-Src [17].

While the cyclopropylamino derivative **2** showed *K*_i_ values approximately 10 µM against both enzymes, the anilino-substituted compound **12**, typed also by the presence of a bromine in R [10], was characterized by a relevant improvement in the inhibitory efficacy against c-Src. Among the phenethylamine derivatives, compound **8** possessed a promising activity against c-Src, but a lower activity against Abl, probably due to the presence of methanethiol group in R_3_ position [19], while compound **10** substituted with a chlorine in R, showed a better profile, acting as a low-micromolar dual inhibitor (*K*_i_ values of 0.22 and 0.31 µM against c-Scr and Abl, respectively). The present enzymatic studies revealed a general improvement in the inhibitory efficacy against c-Src, with *K*_i_ values usually lower than the reference compound **1**. The low-micromolar inhibitory activity against Abl was confirmed and sometimes also improved; only a few exceptions resulted in potential TKI inhibition worse than compound **1**.

#### 3.1.2. Cell Viability Assay

In light of the previous results, further SAR studies were conducted to investigate the antiproliferative activity of the pyrazolo[3,4-*d*]pyrimidine derivatives against four GBM cell lines (U-87, LN18, LN229, and DBTRG) (Table 2).

Compound **1**, previously reported to be active against U-87 cells in the low-micromolar range [10], confirmed its potent antitumor activity against the other GBM cell lines (LN18, LN229, and DBTRG). As shown in Table 2, the cyclopropylamine derivative **2** resulted the least active of the series with only a slight activity against LN18 cells, while compounds **3** and **4**, with a fluorine atom in the ortho and para positions of the benzylamine group, displayed promising IC_50_ values. Interestingly, the best results were obtained with compound **5** (characterized by an additional fluorine atom in the R position with respect to reference compound **1**), which showed low-micromolar activity against all GBM cells tested (concentration vs. cytotoxic effect curves relative to each cell line are reported in Figure S1, Supplementary Materials). Ortho and para fluorobenzylamino derivatives bearing a fluorine (**6**) or chlorine (**7**) atom in the R position presented a slight or significant improvement in anticancer efficacy compared to the corresponding unsubstituted compounds (**3** and **4**) on the N1 side-chain. To further investigate the SAR around the scaffold, substituents such as methanethiol and 4-morpholinoethanethiol moieties were introduced in R_3_ position leading to compounds **8** and **9**, respectively. The phenethylamine derivative **8** demonstrated a mild ability to act as an antiproliferative agent, especially against LN18 cells, while the efficacy of compound **9** was reduced in the other GBM cell lines. Compound **10** followed the same trend as compound **8**, proving to be a valuable antiproliferative agent with encouraging IC_50_ values. Lastly, if compound **11** was a weak cytotoxic agent, the introduction of the aniline moiety in R_2_ (**12**) produced a potent compound active against U-87 cells [10], but was ineffective against the other GBM cell lines. Taken together, the present results strongly suggest that our compounds are promising anticancer candidates toward GBM. Compound **1** and the two most encouraging derivatives (**5** and **7**), endowed with antiproliferative activity comparable to that of reference one, were further investigated for their safety profile by assessing their activity in two different nontumoral, immortalized healthy cells (FIBRO 2-93 and HaCaT). Except for compound **7** which affected the viability of both healthy cell lines (CC_50_ values of 28.6 and 37.8 µM on FIBRO 2-93 and HaCaT, respectively), compounds **1** and **5** were characterized by an optimal safety profile (CC_50_ ~87 µM for compound **1** and >90 µM for compound **5**, i.e., 10 times higher than their IC_50_), thus suggesting a reassuring ability to distinguish between tumoral and healthy cells.

### 3.2. In Vitro ADME Evaluation

To assess the capability of the selected pyrazolo[3,4-*d*]pyrimidine derivatives to reach their target in vivo, the in silico and in vitro ADME properties were assessed for all compounds (Table 3). Our research group was efficiently involved in the attempt to overtake the suboptimal solubility of these compounds with prodrugs [10] or formulations such as albumin nanoparticles [27], cyclodextrins [28], and liposomes [27]. For this reason, we decided to analyze this property using a computational approach followed by proper in vitro investigations. The QikProp software confirmed the hydrophobicity of the derivatives. All compounds were characterized by suboptimal LogS values which could limit their in vivo bioavailability depending on the solvation rate. Nevertheless, the entire series of pyrazolo[3,4-*d*]pyrimidines had the optimal ability to cross the GI membrane and, most importantly, the BBB, as demonstrated by PAMPA assays. Similar to the reference compound **1**, previously reported to show high passive permeability across membranes, compounds **3**, **4**, **5**, **6**, **7**, **10**, and **11** were characterized by high apparent permeability values, never lower than 10 cm/s × 10^−6^, and limited percentages of membrane retention.

The in silico and in vitro results confirmed the well-known suboptimal aqueous solubility characterizing the pyrazolo[3,4-*d*]pyrimidine derivatives, as well as the satisfactory ability to efficiently overcome membranes (especially the BBB) without remaining entrapped in the phospholipidic bilayer. Regarding the metabolic stability in the presence of HLMs, the principal metabolites were identified because of dechlorination (M_1_ and M_4_), oxidation (M_2_), and the loss of the groups bearing the amine moiety in R_2_ (M_3_). The percentage of metabolite formation was calculated as previously reported [18]. With respect to reference compound **1**, most of the derivatives resulted in higher metabolic stability, around 90% or more (**2**, **4**, **7**, **8**, **9**, **11**, and **12**), when incubated in presence of HLMs. Compound **6** underwent massive metabolization with the formation of more than 20% of metabolite M_1_, resulting definitively worse than the reference compound **1**. Lastly, compounds **3**, **5,** and **10** were characterized by improved metabolic stability of approximately 80–85%. As a general conclusion, metabolic stability studies highlighted the tendency of selected compounds to lead to the massive formation of metabolite M_1_ (M − HCl + O), followed by the loss of the group bearing the NH moiety in R_2_ (M_3_).

### 3.3. Irreversible Cytotoxic Effect on LN229 Cell Death

**Figure 1:**
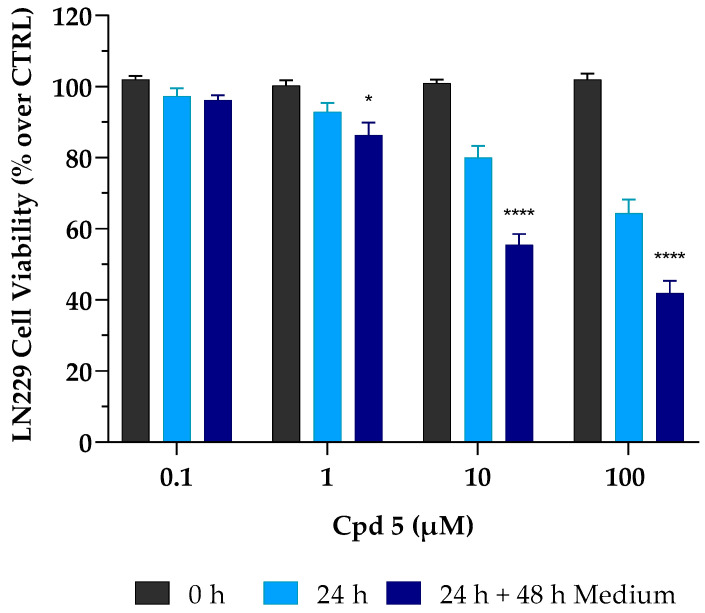
Irreversible cytotoxic effect of compound **5** (Cpd **5**) on LN229 GBM cell line. These were treated with the compound for 24 h (24 h, light-blue column) and then incubated for 48 h with fresh, drug-free medium (24 h + 48 h medium, dark-blue column). Afterward, cell viability was assessed using the MTT assay. Values are the means ± SD of *n* = 3 independent experiments run in triplicate. * *p* < 0.05, **** *p* < 0.001 vs. 24 h at the same concentration (ANOVA followed by Tukey’s multiple comparison test).

Results so far obtained highlighted that compound **5** is a dual inhibitor, endowed with low-micromolar activity against four different GBM cell lines, an optimal safety profile toward healthy cells, and promising ADME properties, particularly a high BBB permeability that represents the Achilles heel of many GBM candidates. These characteristics strongly led us to further investigate its anticancer activity. In the case of cancer cells, it is mandatory to investigate whether a cell line may (or may not) be able to restart its proliferative activity upon drug treatment. It is crucial to demonstrate the presence of the so-called “point of no return”, a limit line between cell injury and cell death, whereby surpassing this point drives irreversible damage. Thus, to better elucidate the biological effect and the mechanism of compound **5**-mediated cell death, further investigations were performed on the LN229 cell line. To check whether the growth inhibitory effect was reversible or irreversible, LN229 cells were initially treated with compound **5** for 24 h; after that, cells were carefully washed to remove the treatment and were cultured for a further 48 h in a complete drug-free medium. At the timepoint of 72 h, as described above, the MTT assay was used to assess cell viability. As reported in Figure 1, compound **5** caused an irreversible cytotoxic effect. This trend was emphasized at the concentrations of 10 and 100 µM with a further decrease in cell viability (~−30% for both concentrations) after 24 h of treatment, followed by 48 h of incubation without compound **5**.

Interestingly, it is important to note that, after 24 h of treatment, cytotoxic effects occurring at both 1 and 10 µM, although moderate (~−10% and −20%, respectively), were immediately irreversible, as cell viability was not restored after withdrawal of the drug. This might prove to be very useful to support a full cytotoxic effect achieved in the low µM range after 72 h of treatment.

### 3.4. Cell-Cycle Analysis

**Figure 2:**
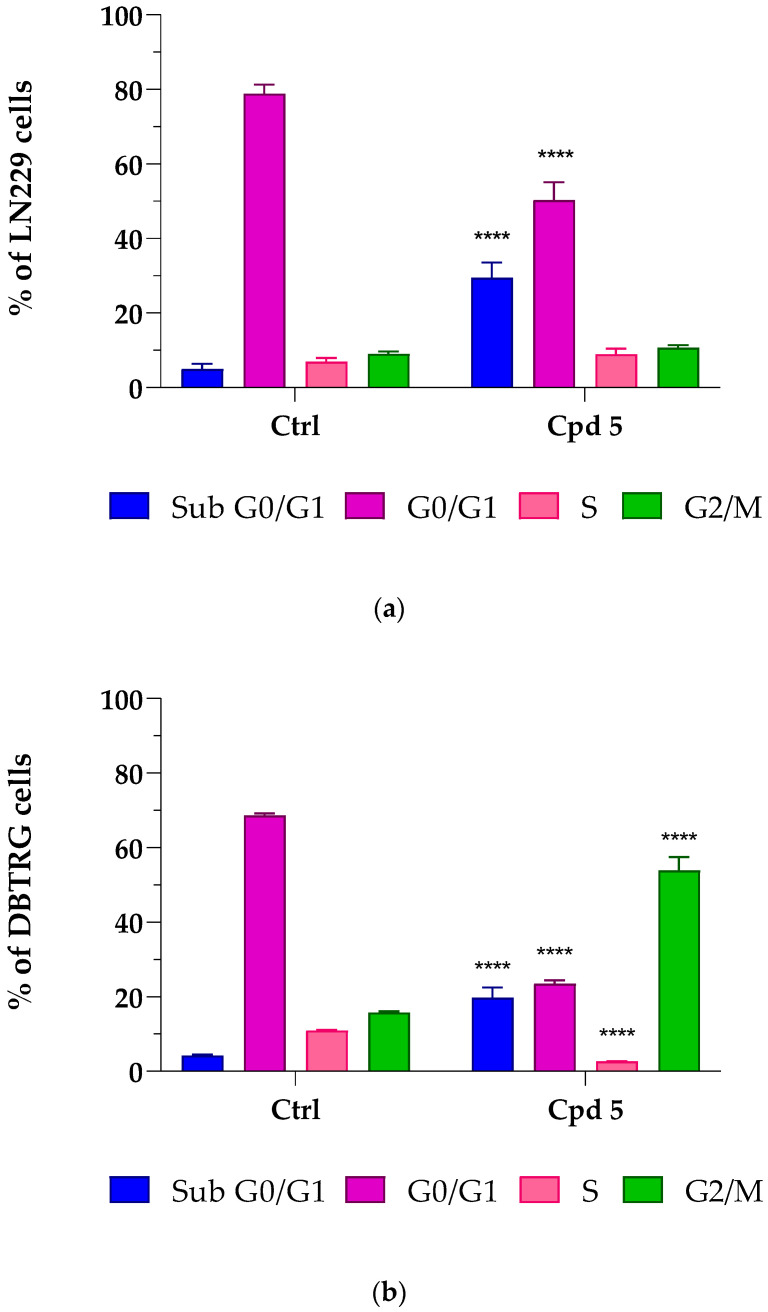
Compound **5**-mediated effects on the glioblastoma LN229 (**a**) and DBTRG (**b**) cell cycle. Cells were treated for 72 h at the compound **5** IC_50_ concentrations, i.e., 6.9 µM for LN229 and 8.5 µM for DBTRG. Percentages of cells in sub G0/G1 (Blue), G0/G1 (Purple), S (Pink), and G2/M (Green) phases are reported as the means ± SD of at least three independent experiments run in triplicate. **** *p* < 0.0001 vs. ctrl, same cell-cycle phase (unpaired Student *t*-test).

To further investigate whether compound **5**-mediated cell death involved some characteristics of apoptosis such as loss of DNA due to DNA fragmentation, as well as effects on cell-cycle progression, flow cytometry-mediated cell-cycle analyses were conducted by treating LN229 and DBTRG cells for 72 h with compound **5** at its IC_50_ concentrations. As reported in Figure 2a, a relevant accumulation of LN229 hypodiploid cells in the sub G0/G1 phase was observed (+32.1%, *p* < 0.0001 vs. control), which was accompanied by a proportional reduction in those in the G0/G1 phase (−32.4%, *p* < 0.0001 vs. control), suggesting that compound **5** induced apoptotic-mediated cell death. No significant differences were detected in the percentage of cells in the S and G2/M phases. As reported in Figure 2b, a significant accumulation of hypodiploid cells was also found in the sub G0/G1 area in DBTRG cells (+15.6%, *p* < 0.0001 vs. control) (see exemplificative histograms of cell-cycle analysis reported in Figure S4, Supplementary Materials), thus suggesting that, in the presence of compound **5**, both LN299 and DBTRG cells respond by initiating programmed cell death. This hypothesis was also supported by the analysis at phase-contrast microscopy, which highlighted significant morphological alterations, including a tendency to round up and detach from the culture plate, shrinkage, loss of contact with adjacent cells, membrane blebbing, and formation of apoptotic bodies (see Figures S2 and S3, Supplementary Materials). The increased number of cells with DNA fragmentation was accompanied by an arrest of DBTRG cells in the G2/M phase (+38.1%, *p* < 0.0001 vs. control).

A cell-cycle completion is essential for proliferation, and cell apoptosis often results from cell-cycle arrest [29]. To complete the cell cycle, a successful G2/M phase is crucial, and uncontrolled cell proliferation might result from imbalanced cell-cycle regulation, which is a hallmark of cancer cells, including GBM [30,31]. Several anticancer drugs have been found to induce G2/M cell-cycle arrest and apoptosis in different GBM cell lines, effectively suppressing proliferation [32,33]. Moreover, the observation that, upon compound **5** treatment, G2/M phase arrest was observed in DBTRG, but not in LN299 cells can be explained by considering that these GBM cell lines possess a diverse genetic background and, in turn, possibly different mechanisms leading to tumor development [34], which might impact the cells’ capacity to respond to drugs. Taken together, results from cell-cycle analysis, showing an increased population in cells with a reduced DNA content (LN299 and DBTRG) and G2/M arrest (DBTRG), suggest the possibility of an apoptotic-mediated cell death caused by compound **5**. As types of cell death other than apoptosis might result in loss of DNA appearing in the sub G0/G1 region, the possibility that other mechanisms may drive compound **5** effects cannot be ruled out.

### 3.5. T-Scratch Assay

**Figure 3:**
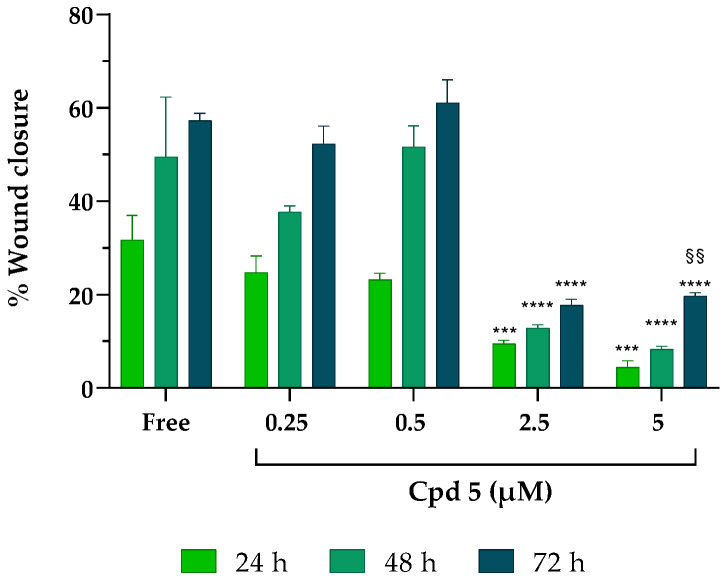
Effect of compound **5** on cancer cell migration. After scratching, LN229 cells were treated with increasing concentrations of compound **5** for 24–48–72 h. Results were obtained from *n* = 3 independent experiments run in triplicate; values are reported as the means ± SD. Two-way ANOVA followed by Tukey’s multiple comparison test was performed to determine statistical significance. 2.5 µM and 5 µM: *** *p* < 0.001, **** *p* < 0.0001 vs. free at the same timepoints; 5 µM at 72 h: §§ *p* < 0.01 vs. the same concentration at 24 h.

Glioblastoma is a malignant primary brain tumor strongly characterized by aggressive invasion of the surrounding cerebral tissues that obstacles surgery and target therapies. Although gliomas rarely metastasize out of the brain tissue, the massive infiltration of tumor cells along blood vessels, with matter tracts and the subarachnoid space is a common hallmark of this type of cancer [35,36]. Therefore, studying the effect of drugs on cell migration is of primary importance; to fulfill this task, we decided to use an easy, low-cost, and reproducible T-scratch assay. The overall ability of compound **5** to influence GBM cell migration was assessed in LN229 cells (Figure 3).

While, in free samples and in the presence of the lowest concentrations of the inhibitor (0.25 and 0.5 µM), the closure of the monolayer significantly increased after 72 h (getting close to 60% of wound closure), at 2.5 and 5 µM, compound **5** suppressed healing, leaving more than 80% of the wound opened at all the timepoints analyzed, suggesting that compound **5** can inhibit the horizontal migration ability of LN229 cells already at the early stage (24 h) of drug stimulation and is sustained over time (72 h), albeit with decreased inhibition rates.

## 4. Conclusions

GBM continues to be a highly aggressive tumor with low expectations of recovery. Today, the standard treatment for GBM is surgical ablation, combined with and/or followed by radiotherapy and chemotherapy. However, such methods are not curative, and patients affected by GBM still have poor prognoses, primarily due to the onset of resistance, often caused by the presence of cancer stem cells [6]. Progressive and increasing interest in developing small-molecule kinase inhibitors has been recorded since the 1980s, with more than 30 new drugs approved by the FDA for the treatment of tumors [37]. In recent years, our research team has created a large library of pyrazolo[3,4-*d*]pyrimidines, capable of inhibiting tyrosine kinases, identified as potent antitumor agents, demonstrating a powerful action both in vitro and in vivo. Thus, moving from our in-house library, a panel of compounds characterized by the pyrazolo[3,4-*d*]pyrimidine scaffold and heterogeneously decorated at N1, C4, and C6 was selected for this study. Compounds were tested against c-Src and Abl, showing promising *K*_i_ values in the micromolar and low-micromolar range. Then, the antiproliferative activity of the compounds was assessed on four GBM cell lines (U-87, LN229, LN18, and DBTRG); for the most active derivatives, a safety profile was established with the healthy fibroblasts FIBRO 2-93 and keratinocytes HaCaT. The ability of the compounds to cross both GI and BBB membranes was assessed in vitro. The optimal passive permeability values suggest that several compounds could efficiently cross the BBB. Moreover, a complete ADME profile was studied for all compounds, revealing comparable solubility but higher metabolic stability with respect to parent compound **1**. Lastly, considering the in vitro biological and pharmacokinetic data, compound **5** was chosen to further characterize its biological effect on LN229 and DBTRG GBM cells. Compound **5** induced irreversible cytotoxicity, significantly reduced cell migration, and increased the number of cells with DNA fragmentation, possibly because of an apoptotic-mediated cell death. Overall, this study led to the identification of a new promising pyrazolo[3,4-*d*]pyrimidine derivative for GBM treatment. Although further investigations are needed to characterize its mechanism of action, as well as to evaluate the in vivo pharmacokinetic profile and efficacy, our present findings demonstrate that compound **5** could represent a new excellent approach to GBM treatment.

## Figures and Tables

**Table 1:** Chemical structures and enzymatic activity (µM) against c-Src and Abl of compounds **1**–**12**.

	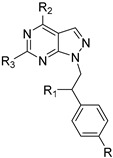				Enzymatic Data	
Compounds	R	R_1_	R_2_	R_3_	c-Src *K*_i_^a^	Abl *K*_i_^a^
**1 ^b^**	H	Cl	NHCH_2_C_6_H_5_	H	3 *	0.37 *
**2**	H	Cl	NH-cyclopropyl	H	11.57 ± 1.08	8.01 ± 0.73
**3 ^c^**	H	Cl	NHCH_2_C_6_H_4_-*o*F	H	0.15 ± 0.02	0.73 ± 0.05 *
**4 ^c^**	H	Cl	NHCH_2_C_6_H_4_-*p*F	H	0.51 ± 0.08	2.00 ± 0.10 *
**5 ^c^**	F	Cl	NHCH_2_C_6_H_5_	H	3.14 ± 0.38	0.44 ± 0.06 *
**6 ^c^**	F	Cl	NHCH_2_C_6_H_4_-*o*F	H	0.07 ± 0.01	0.02 ± 0.01 *
**7 ^d^**	Cl	Cl	NHCH_2_C_6_H_4_-*p*F	H	0.80 ± 0.09	0.08 *
**8 ^e^**	H	Cl	NHCH_2_CH_2_C_6_H_5_	SCH_3_	0.70 *	7.30 *
**9 ^f^**	H	Cl	NHCH_2_C_6_H_5_	SCH_2_CH_2_-4-morpholino	2.90 ± 0.30 *	0.09 ± 0.01 *
**10**	Cl	Cl	NHCH_2_CH_2_C_6_H_5_	H	0.22 ± 0.03	0.31 ± 0.02
**11 ^e^**	H	H	NHCH_2_C_6_H_4_-*m*F	H	0.51 *	4.92 *
**12 ^b^**	Br	Cl	NHC_6_H_5_	H	0.06 *	0.61 *

^a^ Values are the means ± SD of three different experiments run in triplicate. * Compounds previously published. ^b^ Ref. [10]. ^c^ Ref. [16]. ^d^ Ref. [12]. ^e^ Ref. [19]. ^f^ Ref. [17].

**Table 2:** Antiproliferative activity (µM) of compounds **1**–**12** against four GBM cell lines and cytotoxicity (µM) of the most promising ones on nontumoral, healthy FIBRO 2-93 and HaCaT cell lines.

Compounds	IC_50_^a^				CC_50_^b^	
	U-87	LN18	LN229	DBTRG	FIBRO 2-93	HaCaT
**1 ^c^**	2.9 ± 1.1 *	3.6 ± 0.5	3.4 ± 0.8	4.3 ± 0.8	86.9 ± 4.2	88.7 ± 5.7
**2**	NA	49.4 ± 2.8	NA	NA	-	-
**3**	40.2 ± 3.6	27.5 ± 2.1	29.2 ± 2.6	15.1 ± 2.1	-	-
**4**	29.8 ± 2.5	9.0 ± 1.2	NA	68.5 ± 3.8	-	-
**5**	6.8 ± 0.3	10.8 ± 1.4	6.9 ± 0.5	8.5 ± 1.4	91.7 ± 5.3	126.5 ± 6.4
**6**	32.4 ± 2.7	11.7 ± 1.5	33.8 ± 2.3	20.1 ± 1.8	-	-
**7**	22.7 ± 2.3	3.9 ± 0.6	7.1 ± 1.1	14.6 ± 0.9	28.6 ± 3.7	37.8 ± 4.9
**8**	37.9 ± 3.9	5.9 ± 1.1	26.1 ± 2.8	31.2 ± 2.5	-	-
**9**	NA	2.9 ± 0.5	58.2 ± 3.4	59.4 ± 3.7	-	-
**10**	33.9 ± 2.9	6.2 ± 1.0	27.9 ± 2.1	46.5 ± 4.1	-	-
**11**	56.9 ± 3.3	34.1 ± 2.9	58.4 ± 3.0	69.2 ± 3.9	-	-
**12 ^c^**	14.0 ± 1.9 *	NA	NA	NA	-	-

^a^ IC_50_ = drug concentration that caused 50% of cell growth inhibition, ^b^ CC_50_ = concentration that reduced the proliferation of cells by 50%, both determined by measuring the cell viability with the colorimetric MTT assay. Values are the means ± SD of at least three different experiments run in triplicate. * Compounds previously published. ^c^ Ref. [10]. NA means not active (IC_50_ > 100 µM). Empty cells were not determined.

**Table 3:** In silico and in vitro ADME properties of compounds **1**–**12**.

Compounds	QP LogS	GI Papp ^a,b^ (MR%) ^c^	BBB Papp ^a,b^ (MR%) ^c^	Metabolic Stability ^a^ (%)	Metabolite Formation (%) ^d^
**1 ^e^**	−5.715	11.08 *	16.5 *	78.3 *	M_1_ = 14.5 * M_2_ < 0.1 * M_3_ = 2.6 * M_4_ = 4.5 *
**2**	−4.862	6.46 (34.7)	4.96 (55.0)	96.3	M_1_ = 2.1 M_2_ = 1.6
**3**	−6.358	12.4 (7.3)	12.2 (4.8)	80.4	M_1_ = 14.5 M_3_ = 5.1
**4**	−6.784	11.2 (7.8)	10.6 (2.1)	92.2	M_1_ = 5.9 M_2_ = 1.0 M_3_ = 0.9
**5**	−6.511	10.7 (9.2)	11.2 (5.6)	84.2	M_1_ = 10.8 M_2_ = 1.7 M_3_ = 3.3
**6**	−6.955	11.4 (10.1)	12.4 (4.4)	69.9	M_1_ = 21.9 M_2_ = 3.0 M_3_ = 5.2
**7 ^f^**	−7.533	16.6 *	9.6 (4.4)	94.0 *	M_1_ = 4 * M_3_ = 2 *
**8**	−6.207	3.2 (42.7)	5.96 (18.3)	96.6	M_1_ = 1.9 M_2_ = 1.5
**9 ^g^**	−5.6 *	9.1 *	2.6 (6.3)	95 *	M_1_ = 5 *
**10**	−7.589	10.8 (17.2)	11.3 (4.8)	85.9	M_1_ = 10.3 M_3_ = 3.8
**11**	−5.973	10.1 (6.7)	10.8 (5.2)	93.0	M_1_ = 0.4 M_2_ = 6.7
**12 ^e^**	−6.576	6.64 *	13.1 *	96.4 *	-

^a^ Values represent the mean values of three independent experiments run in triplicate. ^b^ Apparent permeability (Papp) reported in cm/s × 10^−6^. ^c^ Membrane retention %. ^d^ M_1_ = M − HCl + O (−36 + 16); M_2_ = M + OH (−16); M_3_ = M—group bearing NH moiety in R_2_; M_4_ = M − Cl + OH (−35 + 17). * Compounds previously published. ^e^ Refs. [10,18]. ^f^ Ref. [12]. ^g^ Ref. [17]. Empty cells were not determined or not found.

## Data Availability

Data are reported in this article and the Supplementary Materials.

## Supplementary Materials

The following supporting information can be downloaded at https://www.mdpi.com/article/10.3390/pharmaceutics15020453/s1, Chemistry; Scheme S1: Chemical Structures of tested compound; Figure S1: Effects of compound **5** on viability of four different GBM cell lines (U-87, LN18, LN229, DBTRG), as well as on two non-tumoral, healthy cells (the fibroblast FIBRO 2-93 cells and the keratinocytes HaCaT cells). Cell viability was expressed as percentage of that of DMSO-treated cells (controls), taken as 100%. IC_50_ and CC_50_ values were calculated by fitting data according to a non-linear regression analysis (sigmoidal log concentration vs. normalized response curve) and reported in Table 2. Each point represents mean ± SD (if not visible, SD was covered by the point); Figure S2: Morphological comparison performed at contrast phase microscopy (scale bar 180 μm) between controls, untreated LN229 cells (panel (a)) and those treated with compound **5** (6.9 µM, 72 h, panel (b)) in which significant morphological alterations, including tendency to round-up, shrinkage, loss of contact with adjacent cells, membrane blebbing and formation of apoptotic bodies are evident. Each photograph was representative of three independent observations; Figure S3: Morphological comparison performed at contrast phase microscopy (scale bar 180 μm) between controls, untreated DBTRG cells (panel (a)) and those treated with compound **5** (8.5 µM, 72h, panel (b)) in which significant morphological changes caused by the drug are evident. Compound **5** caused the cells to lose contact with adjacent cells, cell membrane blebbing and a general tendency to round-up. Each photograph was representative of three independent observations; Figure S4: Exemplificative cell cycle distribution histograms of the LN299 and DBTRG cell lines in control condition (CTRL) and after 72 h of treatment with compound **5** (6.9 µM and 8.5 µM, respectively). Following incubation, the DNA of the cells was stained with propidium iodide and its content was analysed by flow cytometry. The cells were normally distributed in the population of sub G0/G1, G0/G1, S, and G2/M of cell cycle phases in CTRL samples. After 72 h of treatment with compound **5**, an increase in sub G0/G1 cells in both cells lines was observed, while the same compound also caused an arrest the cell cycle in G2/M phase in DBTRG cells; Figure S5: Time-lapse images of a representative T-scratch assay to investigate the effect of compound **5** on GBM cancer cell migration. LN229 cells were seeded, scratched, and finally incubated with increasing concentrations of compound **5** (0.25–0.5–2.5–5 µM) for 24–48–72 h. Scale bar 100 µm.
