# Supplementary material for: Biological Evaluation and In Vitro Characterization of ADME Profile of In-House Pyrazolo[3,4-d]pyrimidines as Dual Tyrosine Kinase Inhibitors Active against Glioblastoma Multiforme
Source: Pharmaceutics. 2023 Jan 30;15(2):453. doi: 10.3390/pharmaceutics15020453 (PMC9966370; doi:10.3390/pharmaceutics15020453)
Supplement: Supplementary file 1 [file pharmaceutics-15-00453-s001.zip › pharmaceutics-2028688-supplementary.pdf]

Supplementary Materials for:

Biological Evaluation and *In Vitro* Characterization of ADME Profile of in-house Pyrazolo[3,4-*d*]Pyrimidines as Dual Tyrosine Kinase Inhibitors Active Against Glioblastoma Multiforme

Federica Poggialini[1], Chiara Vagaggini[1], Annalaura Brai[1], Claudia Pasqualini[1],Emmanuele Crespan[2], Giovanni Maga[2], Lorenzo Botta[3], Francesca Musumeci[4], Maria Frosini[5], Silvia Schenone[4] and Elena Dreassi[1,*]

[1] Department of Biotechnology, Chemistry, and Pharmacy (DBCF), University of Siena, 53100 Siena Italy
[2] Institute of Molecular Genetics~(IGM), CNR "Luigi Luca Cavalli-Sforza", 27100 Pavia, Italy
[3] Department of Ecological and Biological Sciences, University of Tuscia, Via S.C. De Lellis s.n.c., 01100 Viterbo, Italy
[4] Department of Pharmacy, University of Genoa, 16132 Genoa, Italy
[5] Department of Life Sciences, University of Siena, 53100 Siena, Italy
* Correspondence: elena.dreassi@unisi.it; Tel.: +39-0577-234321

Summary

Chemistry 2
Scheme S1. Chemical Structures of compounds 4
Figure S1. IC_{50} and CC_{50} curves for compound 5 5
Figure S2. Effects of compound 5 on LN229 cells morphology 6
Figure S3. Effects of compound 5 on DBTRG cells morphology 7
Figure S4. Cell cycle distribution data 8
Figure S5. T-Scratch Assay 9
References 10

Chemistry

N-benzyl-1-(2-chloro-2-phenylethyl)-1H-pyrazolo[3,4-d]pyrimidin-4-amine (1). White solid, mp 158–160 °C, yield 82%. 1H NMR: δ 4.69–4.80 and 4.93–5.07 (2dd, 2H, CH_2N), 4.82–4.88 (m, 2H, CH_2NH), 5.51–5.59 (m, 2H, CHCl), 7.25–7.51 (m, 10H Ar), 7.85 (s, 1H, H-3), 8.39 (s, 1H, H-6). IR cm^{-1}: 3425 (NH) [1].

1-(2-chloro-2-phenylethyl)-N-cyclopropyl-1H-pyrazolo[3,4-d]pyrimidin-4-amine (2). White solid, mp 194–195 °C, yield 90%. 1H NMR: δ 0.73–0.87 and 0.98–1.10 (2m, 4H, $2CH_2$), 2.88–3.02 (m, 1H, CH), 4.71–4.83 and 4.95–5.08 (2dd, 2H, CH_2N), 5.53–5.62 (m, 1H, CHCl), 6.68 (brs, 1H, NH, disappears with D_2O), 7.25–7.52 (m, 5H Ar), 8.19 (s, 1H, H-3), 8.32 (s, 1H, H-6). IR cm^{-1}: 3410 (NH) [1].

1-(2-Chloro-2-phenylethyl)-N-(4-fluorobenzyl)-1H-pyrazolo[3,4-d]pyrimidin-4-amine (3). White solid, mp 168–169 °C, yield 69%. 1H NMR: δ 4.62–5.01 (m, 4H, CH_2N + CH_2Ar), 5.40–5.54 (m, 1H, CHCl), 6.90–7.44 (m, 9H Ar), 7.80 (s, 1H, H-3), 8.33 (s, 1H, H-6). IR cm^{-1}: 3247 (NH). Anal. ($C_{20}H_{17}N_5ClF$) C, H, N [2].

1-(2-Chloro-2-phenylethyl)-N-(2-fluorobenzyl)-1H-pyrazolo[3,4-d]pyrimidin-4-amine (4). White solid, mp 161-162 °C, yield 70%. 1H NMR: δ 4.61-4.98 (m, 4H, CH_2N + CH_2Ar), 5.41-5.53 (m, 1H, CHCl), 6.94-7.46 (m, 9H Ar), 7.80 (s, 1H, H-3), 8.33 (s, 1H, H-6). IR cm^{-1}: 3249 (NH). Anal. ($C_{20}H_{17}N_5ClF$) C, H, N [2].

N-Benzyl-1-[2-chloro-2-(4-fluorophenyl)ethyl]-1H-pyrazolo[3,4-d]pyrimidin-4-amine (5). White solid, mp 143-144 °C, yield 79%. 1H NMR: δ 4.62-4.95 (m, 4H, CH_2N + CH_2Ar), 5.40-5.51 (m, 1H, CHCl), 6.96-7.40 (m, 9H Ar), 7.77 (s, 1H, H-3), 8.28 (s, 1H, H-6). IR cm^{-1}: 3198 (NH). Anal. ($C_{20}H_{17}N_5ClF$) C, H, N [2].

1-[2-Chloro-2-(4-fluorophenyl)ethyl]-N-(2-fluorobenzyl)-1H-pyrazolo[3,4-d]pyrimidin-4- amine (6). White solid, mp 171-172 °C , yield 66%. 1H NMR: δ 4.63-4.96 (m, 4H, CH_2N + CH_2Ar), 5.40-5.49 (m, 1H, CHCl), 6.97-7.40 (m, 8H Ar), 7.80 (s, 1H, H-3), 8.32 (s, 1H, H-6). IR cm^{-1}: 3203 (NH). Anal. ($C_{20}H_{16}N_5ClF_2$) C, H, N [2].

1-[2-Chloro-2-(4-chlorophenyl)ethyl]-N-(4-fluorobenzyl)-1H-pyrazolo[3,4-d]pyrimidin-4-amine (7). White solid, mp 164-165 °C, yield 80%. 1H NMR: δ 4.64-4.86 (m, 4H, CH_2N + CH_2Ar), 5.41-5.53 (m, 1H, CHCl), 6.90-7.42 (m, 8H Ar), 7.77 (s, 1H, H-3), 8.30 (s, 1H, H-6). IR cm^{-1}: 3244 (NH). Anal. ($C_{20}H_{16}N_5Cl_2F$) C, H, N [2].

1-(2-chloro-2-phenylethyl)-6-methylthio-N-phenethyl-1H-pyrazolo[3,4-d]pyrimidin-4-amine (8). White solid, mp 73–74 °C, yield 76%. 1H NMR: δ 2.59 (s, 3H, CH_3S), 2.98 (q, J = 6.0, 2H, $CH_2C_6H_5$), 3.87 (q, J = 6.0, 2H, CH_2NH), 4.70–4.95 (m, 2H, CH_2N), 5.30 (br s, 1H, NH, disappears with D_2O), 5.50–5.60 (m, 1H, CHCl), 7.19–7.48 (m, 10H Ar), 7.73 (s, 1H, H-3). IR cm^{-1}: 3445 (NH) [3].

N-Benzyl-1-(2-chloro-2-phenylethyl)-6-[(2-morpholin-4-ylethyl)-thio]-1H-pyrazolo[3,4-d]pyrimidin-4-amine (9). White solid (3.56 g, 70%). mp 124-125 °C. 1H NMR: δ 2.50 (t, J = 4.4, 4H, 2CH_2N morph.), 2.64-2.81 (m, 2H, CH_2CH_2S), 3.10-3.38 (m, 2H, CH_2S), 3.58-3.72 (m, 4H, 2CH_2O morph.), 4.53-4.94 (m, 4H, CH_2N + CH_2Ar), 5.40-5.52 (m, 1H, CHCl), 7.12-7.42 (m, 10H Ar), 7.66 (s, 1H, H-3). IR cm^{-1}: 3197 (NH). MS: m/z 509 $[M+1]^+$. Anal. ($C_{26}H_{29}N_6OClS$) C, H, N, S [4].

1-[2-chloro-2-(4-chlorophenyl)ethyl]-N-(2-phenylethyl)-1H-pyrazolo[3,4-d]pyrimidin-4-amine (10). White solid (243 mg, 59%). mp 143-144 °C. 1H NMR (200 MHz, $(CD_3)_2SO$): δ 3.03 (t, J = 7.0, 2H, CH_2Ar), 3.82 (q, J = 7.0, 2H, CH_2NH), 4.70-4.83 and 4.93-5.05 (2m, 2H, CH_2N), 5.53-5.65 (m, 2H, CHCl + NH disappears with D_2O), 7.24-7.55 (m, 9H Ar), 7.87 (s, 1H, H-3), 8.45 ppm (s, 1H, H-6); IR (KBr) cm-1: 3330 (NH). MS: m/z 412 $[M+1]^+$; Anal. calc for $C_{21}H_{19}N_5Cl_2$: C 61.17, H 4.64, N 16.99, found C 61.27, H 4.87, N 17.11 [5].

N-(3-fluorobenzyl)-1-(2-phenylethyl)-1H-pyrazolo[3,4-d]pyrimidin-4-amine (11). White solid (239 mg, 69 %). mp 113-114 °C. 1H NMR: δ 3.15 (t, J = 7.0, 2H, CH_2Ar), 4.56 (t, J = 7.0, 2H, CH_2N), 4.78 (d, 2H, $NHCH_2$), 6.86-7.32 (m, 9H Ar), 7.78 (s, 1H, H-3), 8.27 (s, 1H, H-6). IR cm^{-1}: 3240 (NH). Anal.($C_{20}H_{18}N_5F$) C, H, N. MS: m/z 347 $[M+1]^+$ [6].

1-[2-(4-Bromophenyl)-2-chloroethyl]-N-phenyl-1H-pyrazolo[3,4-d]pyrimidin-4-amine (12). White solid (244 mg, 57%). mp 190-191 °C. 1H NMR: δ 5.02-5.08 (m, 2H, CH_2N), 5.95-6.01 (m, 1H, CHCl), 7.14-7.45 and 7.67-7.82 (2m, 9H Ar), 8.10 (s, 1H, H-3), 8.17 (s, 1H, H-6). IR cm^{-1}: 3301 (NH). MS: m/z 429 $[M+1]^+$. Anal. ($C_{19}H_{15}N_5BrCl$) C, H, N [7].

Scheme S1. Chemical Structures of compounds

Compound 1	Compound 2	Compound 3
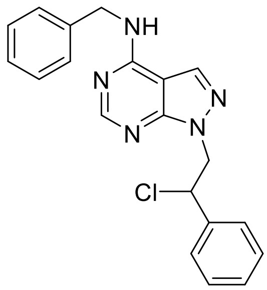	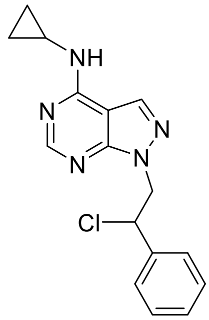	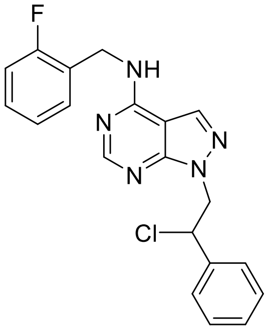
**Compound 4**	**Compound 5**	**Compound 6**
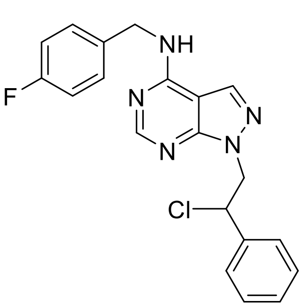	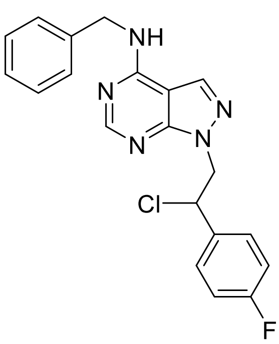	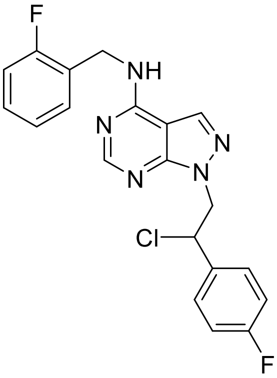
**Compound 7**	**Compound 8**	**Compound 9**
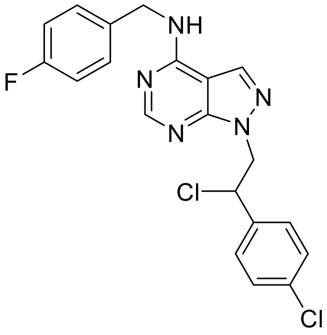	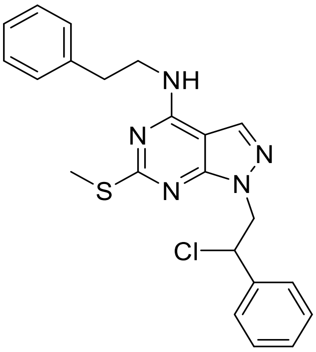	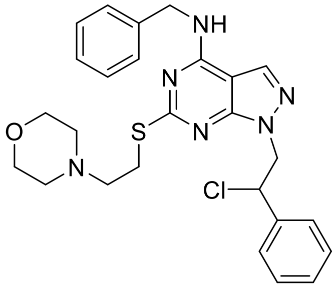
**Compound 10**	**Compound 11**	**Compound 12**
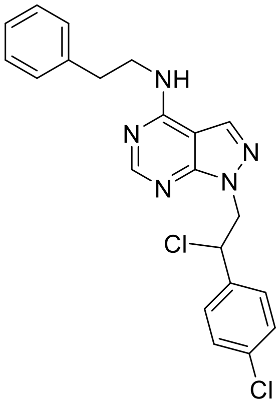	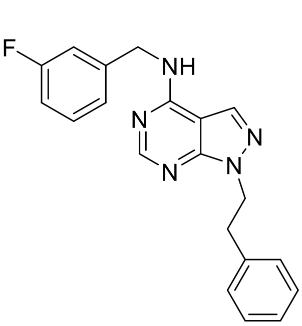	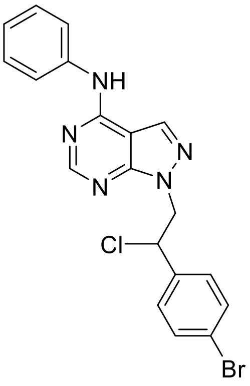

Figure S1. IC_{50} and CC_{50} curves for compound 5

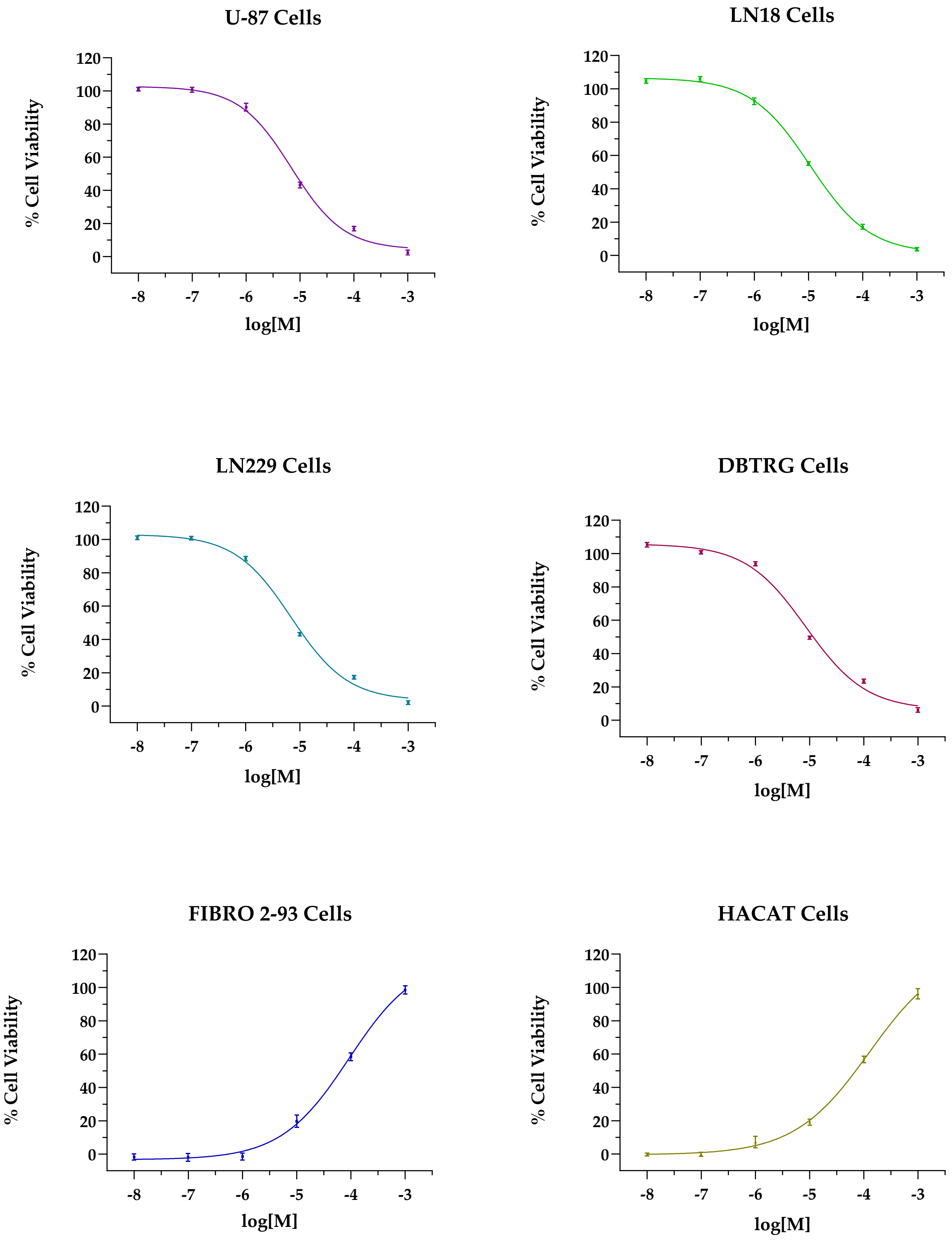

Figure S1. Effects of compound **5** on viability of four different GBM cell lines (U-87, LN18, LN229, DBTRG), as well as on two non-tumoral, healthy cells (the fibroblast FIBRO 2-93 cells and the keratinocytes HaCaT cells). Cell viability was expressed as percentage of that of DMSO-treated cells (controls), taken as 100%. IC_{50} and CC_{50} values were calculated by fitting data according to a non-linear regression analysis (sigmoidal log concentration vs normalized response curve) and reported in Table 2. Each point represents mean ± SD (if not visible, SD was covered by the point).

Figure S2. Effects of compound 5 on LN229 cells morphology

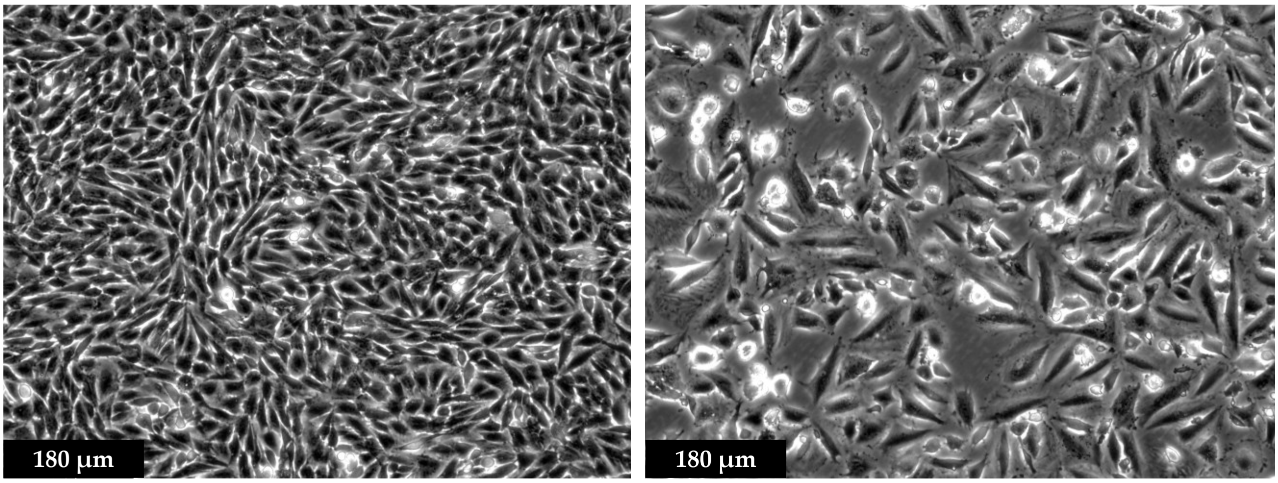

(**a**) (**b**)

Figure S2. Morphological comparison performed at contrast phase microscopy (scale bar 180 μm) between controls, untreated LN229 cells (panel a) and those treated with compound **5** (6.9 μM, 72h, panel b) in which significant morphological alterations, including tendency to round-up, shrinkage, loss of contact with adjacent cells, membrane blebbing and formation of apoptotic bodies are evident. Each photograph was representative of three independent observations**.**

Figure S3. Effects of compound 5 on DBTRG cells morphology

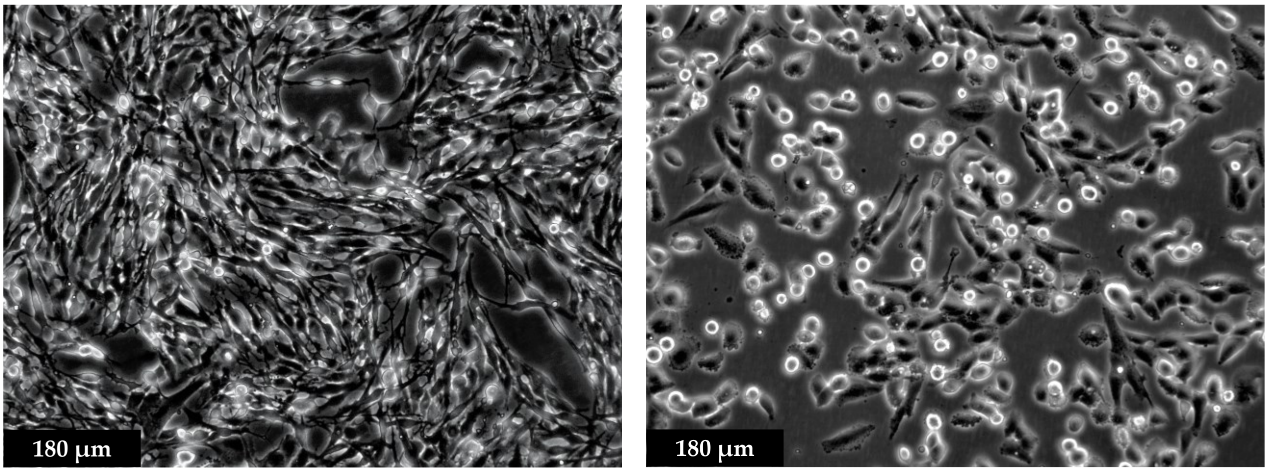

(**a**) (**b**)

Figure S3. Morphological comparison performed at contrast phase microscopy (scale bar 180 µm) between controls, untreated DBTRG cells (panel a) and those treated with compound **5** (8.5 µM, 72h, panel b) in which significant morphological changes caused by the drug are evident. Compound **5** caused the cells to lose contact with adjacent cells, cell membrane blebbing and a general tendency to round-up. Each photograph was representative of three independent observations.

Figure S4. Cell cycle distribution histograms

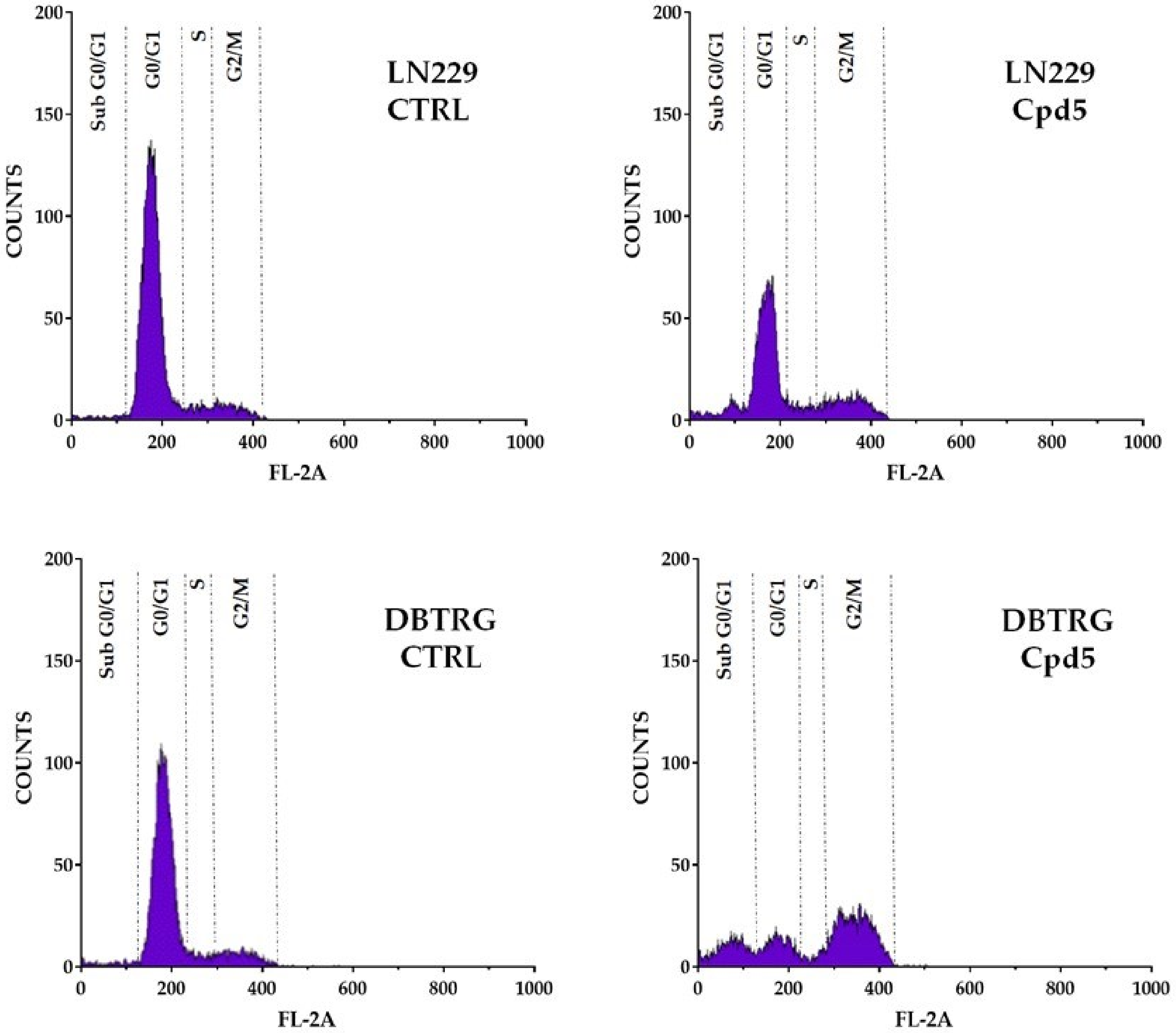

Figure S4. Exemplificative cell cycle distribution histograms of the LN299 and DBTRG cell lines in control condition (CTRL) and after 72 hrs of treatment with compound **5** (6.9 µM and 8.5 µM, respectively). Following incubation, the DNA of the cells was stained with propidium iodide and its content was analysed by flow cytometry. The cells were normally distributed in the population of sub G0/G1, G0/G1, S, and G2/M of cell cycle phases in CTRL samples. After 72 hrs of treatment with compound **5**, an increase in sub G0/G1 cells in both cells lines was observed, while the same compound also caused an arrest the cell cycle in G2/M phase in DBTRG cells.

Figure S5. T-Scratch Assay

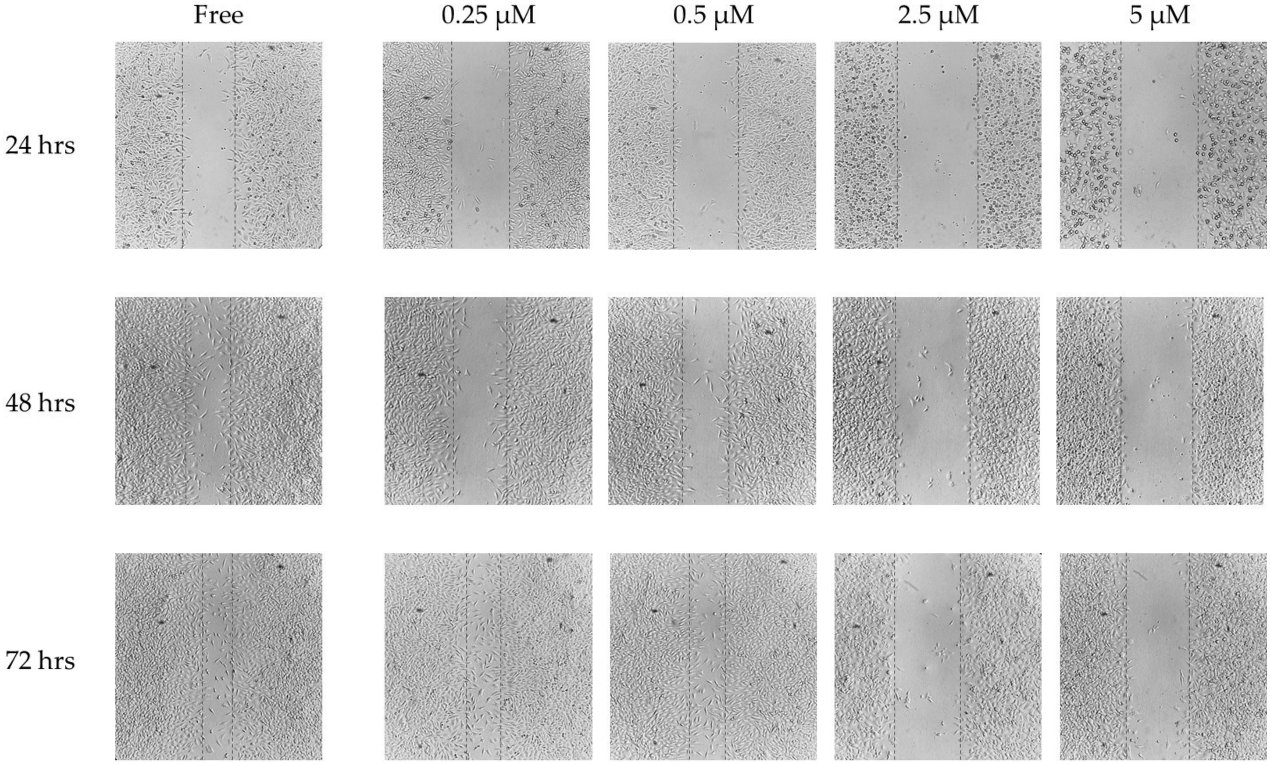

Figure S5. Time-lapse images of a representative T-scratch assay to investigate the effect of compound **5** on GBM cancer cell migration. LN229 cells were seeded, scratched, and finally incubated with increasing concentrations of compound **5** (0.25-0.5-2.5-5 μM) for 24-48-72 hrs. Scale bar 100 μm.

References

(1) Schenone, S.; Bruno, O.; Bondavalli, F.; Ranise, A.; Mosti, L.; Menozzi, G.; Fossa, P.; Donnini, S.; Santoro, A.; Ziche, M.; Manetti, F.; Botta, M. Antiproliferative Activity of New 1-Aryl-4-Amino-1H-Pyrazolo[3,4-d]Pyrimidine Derivatives toward the Human Epidermoid Carcinoma A431 Cell Line. *Eur. J. Med. Chem.* **2004**, *39* (11), 939–946. https://doi.org/10.1016/j.ejmech.2004.07.010.

(2) Manetti, F.; Brullo, C.; Magnani, M.; Mosci, F.; Chelli, B.; Crespan, E.; Schenone, S.; Naldini, A.; Bruno, O.; Trincavelli, M. L.; Maga, G.; Carraro, F.; Martini, C.; Bondavalli, F.; Botta, M. Structure-Based Optimization of Pyrazolo[3,4- d]Pyrimidines as Abl Inhibitors and Antiproliferative Agents toward Human Leukemia Cell Lines. *J. Med. Chem.* **2008**, *51* (5), 1252–1259. https://doi.org/10.1021/jm701240c.

(3) Schenone, S.; Bruno, O.; Bondavalli, F.; Ranise, A.; Mosti, L.; Menozzi, G.; Fossa, P.; Manetti, F.; Morbidelli, L.; Trincavelli, L.; Martini, C.; Lucacchini, A. Synthesis of 1-(2-Chloro-2-Phenylethyl)-6-Methylthio-1H-Pyrazolo[3,4-d]Pyrimidines 4-Amino Substituted and Their Biological Evaluation. *Eur. J. Med. Chem.* **2003**, *39*, 153–160. https://doi.org/10.1016/j.ejmech.2003.11.007.

(4) Radi, M.; Dreassi, E.; Brullo, C.; Crespan, E.; Tintori, C.; Bernardo, V.; Valoti, M.; Zamperini, C.; Daigl, H.; Musumeci, F.; Carraro, F.; Naldini, A.; Filippi, I.; Maga, G.; Schenone, S.; Botta, M. Design, Synthesis, Biological Activity, and ADME Properties of Pyrazolo[3,4- d]Pyrimidines Active in Hypoxic Human Leukemia Cells: A Lead Optimization Study. *J. Med. Chem.* **2011**, *54* (8), 2610–2626. https://doi.org/10.1021/jm1012819.

(5) Zamperini, C.; Dreassi, E.; Vignaroli, G.; Radi, M.; Dragoni, S.; Schenone, S.; Musumeci, F.; Valoti, M.; Antiochia, R.; Botta, M. CYP-Dependent Metabolism of Antitumor Pyrazolo[3,4-d]Pyrimidine Derivatives Is Characterized by an Oxidative Dechlorination Reaction. *Drug Metab. Pharmacokinet.* **2014**, *29* (6), 433–440. https://doi.org/10.2133/dmpk.DMPK-13-RG-094.

(6) Radi, M.; Brullo, C.; Crespan, E.; Tintori, C.; Musumeci, F.; Biava, M.; Schenone, S.; Dreassi, E.; Zamperini, C.; Maga, G.; Pagano, D.; Angelucci, A.; Bologna, M.; Botta, M. Identification of Potent C-Src Inhibitors Strongly Affecting the Proliferation of Human Neuroblastoma Cells. *Bioorg. Med. Chem. Lett.* **2011**, *21* (19), 5928–5933. https://doi.org/10.1016/j.bmcl.2011.07.079.

(7) Radi, M.; Tintori, C.; Musumeci, F.; Brullo, C.; Zamperini, C.; Dreassi, E.; Fallacara, A. L.; Vignaroli, G.; Crespan, E.; Zanoli, S.; Laurenzana, I.; Filippi, I.; Maga, G.; Schenone, S.; Angelucci, A.; Botta, M. Design, Synthesis, and Biological Evaluation of Pyrazolo[3,4- d]Pyrimidines Active in Vivo on the Bcr-Abl T315I Mutant. *J. Med. Chem.* **2013**, *56* (13), 5382–5394. https://doi.org/10.1021/jm400233w.
